# Supplementary material for: The effect of liver enzymes on body composition: A Mendelian randomization study
Source: PLoS One. 2020 Feb 11;15(2):e0228737. doi: 10.1371/journal.pone.0228737 (PMC7012438; doi:10.1371/journal.pone.0228737)
Supplement: S1 Table — (DOCX) [file pone.0228737.s001.docx]

S1 Table. Baseline characteristics of the participants who were included (n=3,455) and excluded (n=4,872) in the analyses of the Hong Kong’s “Children of 1997” birth cohort, Hong Kong, China, 1997 to 2016

| Characteristics | | Included (n=3,455) | | Excluded (n=4,872) | | p-value^a^ | Cohen effect size^b^ |
| --- | --- | --- | --- | --- | --- | --- | --- |
|  |  |  |  |  |  |  |  |
|  |  | n | Mean (SD) / % | n | Mean (SD) / % |  |  |
| Sex | | 3455 |  | 4872 |  | <0.001 | 0.08 |
|  | Female | 1716 | 49.67% | 2197 | 45.09% |  |  |
|  | Male | 1739 | 50.33% | 2610 | 53.57% |  |  |
|  | Unknown | 0 |  | 65 | 1.33% |  |  |
| Second-hand and maternal smoking exposure | | 3455 |  | 4872 |  | <0.001 | 0.09 |
|  | None | 943 | 27.29% | 1232 | 25.29% |  |  |
|  | Prenatal second-hand smoking | 1278 | 36.99% | 1522 | 31.24% |  |  |
|  | Postnatal second-hand smoking | 961 | 27.81% | 1493 | 30.64% |  |  |
|  | Maternal smoking | 128 | 3.70% | 275 | 5.64% |  |  |
|  | Unknown | 145 | 4.20% | 350 | 7.18% |  |  |
| Highest parental education levels | | 3455 |  | 4872 |  | <0.001 | 0.12 |
|  | Grade<=9 | 989 | 28.63% | 1478 | 30.34% |  |  |
|  | Grades 10-11 | 1489 | 43.10% | 1958 | 40.19% |  |  |
|  | Grades>=12 | 961 | 27.81% | 1222 | 25.08% |  |  |
|  | Unknown | 16 | 0.46% | 214 | 4.39% |  |  |
| Highest parental occupation | | 3455 |  | 4872 |  | <0.001 | 0.07 |
|  | Ⅰ(unskilled) | 99 | 2.87% | 140 | 2.87% |  |  |
|  | Ⅱ(semiskilled) | 285 | 8.25% | 441 | 9.05% |  |  |
|  | Ⅲ (semiskilled) | 504 | 14.59% | 711 | 14.59% |  |  |
|  | Ⅲ (nonmanual skilled) | 879 | 25.44% | 1167 | 23.95% |  |  |
|  | Ⅳ (managerial) | 439 | 12.71% | 683 | 14.02% |  |  |
|  | Ⅴ(professional) | 797 | 23.07% | 917 | 18.82% |  |  |
|  | Unknown | 452 | 13.08% | 813 | 16.69% |  |  |
| Household income per head at recruitment | | 3455 |  | 4872 |  | <0.001 | 0.07 |
|  | First quintile | 572 | 16.56% | 879 | 18.04% |  |  |
|  | Second quintile | 616 | 17.83% | 868 | 17.82% |  |  |
|  | Third quintile | 618 | 17.89% | 811 | 16.65% |  |  |
|  | Fourth quintile | 631 | 18.26% | 788 | 16.17% |  |  |
|  | Fifth quintile | 646 | 18.70% | 794 | 16.30% |  |  |
|  | Unknown | 372 | 10.77% | 732 | 15.02% |  |  |
| Type of housing at recruitment | | 3455 |  | 4872 |  | <0.001 | 0.08 |
|  | Public | 1445 | 41.82% | 2131 | 43.74% |  |  |
|  | Subsidized home ownership scheme | 545 | 15.77% | 580 | 11.90% |  |  |
|  | Private | 1360 | 39.36% | 1885 | 38.69% |  |  |
|  | Unknown | 105 | 3.04% | 276 | 5.67% |  |  |

a Two-side *P*-value from chi-square tests

b Cohen effect sizes are usually categorized into 3 levels, Chi-square tests for categorical variables: 0.10 for small. 0.30 for medium, 0.50 for large. For categorical variables, Cohen’s w effect size is calculated as $w=\sqrt{{\chi2}/N}$ where N is the total count of the included and excluded participants.
