# Supplementary material for: The effect of liver enzymes on body composition: A Mendelian randomization study
Source: PLoS One. 2020 Feb 11;15(2):e0228737. doi: 10.1371/journal.pone.0228737 (PMC7012438; doi:10.1371/journal.pone.0228737)
Supplement: S2 Table — (DOCX) [file pone.0228737.s002.docx]

S2 Table. Single nucleotide polymorphisms (SNPs) with potential pleiotropic effects other than via the specific liver enzyme from Ensembl, GWAS Catalog, and potential confounders from UK Biobank

| Liver Enzyme | SNPs | Location | Gene nearby | Phenotype, disease, and trait -Ensembl | Phenotype, disease, and trait - GWAS Catalog | Potential confounders with Bonferroni correction^a^ – UK Biobank | Potential pleiotropy | Potentially confounded |
| --- | --- | --- | --- | --- | --- | --- | --- | --- |
| ALT | rs10883437 | 10q24 | *CPN1* | - | - | - | - | - |
| ALT | rs2954021 | 8q24 | *TRIB1* | HDL, LDL, TC, ALP, Lymphocyte percentage of white cells, Neutrophil percentage of white cells, Response to fenofibrate (triglyceride levels) | Triglyceride levels, ALP, LDL | Alcohol intake frequency (2.57E-5), Height (3.54E-11), | + | + |
| ALT | rs6834314 | 4q22 | *HSD17B13, MAPK10* | - | - | - | - | - |
| ALT | rs738409 | 22q13 | *PNPLA3, SAMM50* | Nonalcoholic fatty liver disease(NFLD), Cirrhosis (alcohol related) | Cirrhosis (alcohol related), Nonalcoholic fatty liver disease | - | - | - |
| ALP | rs10819937 | 9q21 | *ALDOB, C9orf125* | - | - | - | - | - |
| ALP | rs16856332 | 2q24 | *ABCB11* | - | - | - | - | - |
| ALP | rs174601 | 11q12 | *C11orf10, FADS1, FADS2* | Blood metabolite levels, TC, Gondoic acid (20:1n-9) levels, HDL, Red blood cell fatty acid levels, Trans fatty acid levels | Gondoic acid levels, Trans fatty acid levels, Red blood cell fatty acid levels, Blood metabolite levels | Height (2.35E-10) | - | + |
| ALP | rs1883415 | 6p22 | *ALDH5A1, GPLD1* | - | - | - | - | - |
| ALP | rs1976403 | 1p36.12 | *ALPL, NBPF3* | - | - | - | - | - |
| ALP | rs2236653 | 11q.24 | *ST3GAL4* | - | - | Height (4.86E-5) | - | + |
| ALP | rs281377 | 19q13 | *FUT2* | Resting metabolic rate | Yeast infection, Resting metabolic rate | Alcohol intake frequency (2.52E-7), Alcohol intake verse 10 years previously (5.44E-5), Menache (4.46E-4), Height (1.45E-5) | + | + |
| ALP | rs2954021 | 8q24 | *TRIB1* | TC, HDL, LDL, ALT, Lymphocyte percentage of white cells, Neutrophil percentage of white cells, Response to fenofibrate (triglyceride levels) | Triglyceride levels, ALT, LDL | Alcohol intake frequency (2.57E-5), Height (3.54E-11), | + | + |
| ALP | rs314253 | 17p13 | *ASGR1, DLG4* | TC, LDL | LDL cholesterol levels, Total cholesterol | - | - | - |
| ALP | rs579459 | 9q34 | *ABO* | Blood metabolite ratios, C-reactive protein levels, TC, Coronary Artery Disease, Ischemic stroke, Large artery stroke, E-Selectin, LDL, Red blood cell count, Red blood cell traits, Soluble E-selectin levels, Soluble levels of adhesion molecules, Urinary metabolites (H-NMR features), | Glycated hemoglobin levels, Total cholesterol, LDL, Soluble levels of adhesion molecules, Red blood cell count, Urinary metabolites (H-NMR features), Coronary artery disease, Coronary artery disease or large artery stroke, Coronary artery disease or ischemic stroke, Coronary heart disease, Red blood cell traits, Blood metabolite ratios | Height (7.46E-6) | + | + |
| ALP | rs6984305 | 8p23 | *PPP1R3B* | TC, HDL | - | Current tobacco smoking (1.12E-4) | - | + |
| ALP | rs7186908 | 16q22 | *HPR, PMFBP1* | - | - | - | - | - |
| ALP | rs7267979 | 20p11 | *ABHD12,GINS1, PYGB* | - | - | - | - | - |
| ALP | rs7923609 | 10q21 | *JMJD1C, NRBF2* | Educational attainment | Educational attainment | Menache (2.95E-8), Height (1.77E-11), Voice broke (7.33E-5) | - | + |
| GGT | rs10513686 | 3q26 | *SLC2A2* | - | - | - | - | - |
| GGT | rs1076540 | 22q11.21 | *MICAL3* | - | - | - | - | - |
| GGT | rs10908458 | 1q21 | *DPM3, EFNA1, PKLR* | - | - | Past tabacco smoking (3.74E-5) | - | + |
| GGT | rs12145922 | 1p22 | *CCBL2, PKN2* | - | - | Height (2.58E-35), | - | + |
| GGT | rs1260326 | 2p23 | *C2orf16, GCKR* | Blood metabolite levels, C-reactive protein levels, Triglyceride levels, Caffeine metabolism (plasma 1,7-dimethylxanthine (paraxanthine) to 1,3,7-trimethylxanthine (caffeine) ratio), Cardiovascular disease risk factors, TC, Chronic kidney disease, Coffee consumption, Crohn's disease, Fasting Glucose (More seen in http://www.ensembl.org) | Alcohol consumption, Triglyceride, Crohn's disease, Inflammatory bowel disease, Plasma lactate levels, Hypertriglyceridemia, Renal overload goutBlood metabolite levels, Gout, Non-albumin protein levels, Two-hour glucose challenge (More could be assessed in https://www.ebi.ac.uk/gwas/search?query=rs1260326 ) | Alcohol intake frequency (1.28E-43), Alcohol intake verse 10 years previously (1.54E-12), Height (7.79E-22), Voice broke (3.93E-4) | + | + |
| GGT | rs12968116 | 2q37 | *ATP8B1* | Body Height, Familial Intrahepatic Cholestasis | - | - | + | - |
| GGT | rs13030978 | 2q12 | *MYO1B, STAT4* | - | - | - | - | - |
| GGT | rs1335645 | 1p13 | *CEPT1* | - | - | - | - | - |
| GGT | rs1497406 | 1p36.13 | *RSG1, EPHA2* | - | - | Height (3.91E-5), | - | + |
| GGT | rs17145750 | 7q11 | *MLXIPL* | Metabolite levels (lipoprotein measures), Platelet Count | Platelet count, Metabolite levels (lipoprotein measures) | Alcohol intake frequency (5.76E-7), Alcohol intake verse 10 years previously (4.70E-5) | - | + |
| GGT | rs2073398 | 22q11.23 | *GGT1, GGTLC2* | - | - | - | - | - |
| GGT | rs2140773 | 2q37 | *EFHD1, LOC100129166* | - | - | - | - | - |
| GGT | rs2739330 | 22q11.23 | *DDT, DDTL, GSTT1, GSTT2B, MIF* | - | - | - | - | - |
| GGT | rs339969 | 15q21 | *RORA* | - | - | - | - | - |
| GGT | rs4074793 | 5q11 | *ITGA1* | - | - | - | - | - |
| GGT | rs4503880 | 18q21.32 | *NEDD4L* | - | - | - | - | - |
| GGT | rs4547811 | 4q31 | *ZNF827* | - | - | - | - | - |
| GGT | rs4581712 | 16q23 | *DYNLRB2* | - | - | - | - | - |
| GGT | rs516246 | 16q23 | *FUT2* | TC, Crohn's disease (time to surgery), Inflammatory bowel disease, Obesity-related traits | Crohn's disease, Inflammatory bowel disease, Obesity-related traits | Alcohol intake frequency (1.58E-9), Alcohol intake verse 10 years previously (4.40E-7), Menache (1.09E-4), Height (2.88E-7) | + | + |
| GGT | rs6888304 | 5p15 | *CDH6* | - | - | - | - | - |
| GGT | rs7310409 | 12q24 | *HNF1A, C12orf27* | C-reactive protein, Pancreatic Cancer, Pancreatic Neoplasms | Pancreatic cancer, C-reactive protein | Menache (5.51E-7), Voice broke (1.04E-5) | - | + |
| GGT | rs754466 | 10q23 | *DLG5* | - | - | Height (2.57E-17) | - | + |
| GGT | rs8038465 | 15q23 | *CD276* | - | - | - | - | - |
| GGT | rs9296736 | 6p12 | *MLIP* | - | - | - | - | - |
| GGT | rs944002 | 14q32 | *EXOC3L4* | Mean platelet volume | Mean platelet volume | - | - | - |
| GGT | rs9913711 | 17q24 | *FLJ37644, SOX9* | - | - | - | - | - |

ALT: alanine aminotransferase; ALP: alkaline phosphatase; GGT: gamma glutamyltransferase

TC: total cholesterol; HDL: high density lipoprotein cholesterol; LDL: low density lipoprotein cholesterol

a *P*-value with Bonferroni correction for ALT, ALP and GGT are 0.001, 0.0003 and 0.0001
