# Supplementary material for: The effect of liver enzymes on body composition: A Mendelian randomization study
Source: PLoS One. 2020 Feb 11;15(2):e0228737. doi: 10.1371/journal.pone.0228737 (PMC7012438; doi:10.1371/journal.pone.0228737)
Supplement: S3 Table — (DOCX) [file pone.0228737.s003.docx]

S3 Table. Characteristics of palindromic single nucleotide polymorphisms (SNPs) in the exposure and outcome genome-wide association studies

| Phenotype | SNP | Effect Allele | Other Allele | EAF_Exposure | EAF_Outcome |
| --- | --- | --- | --- | --- | --- |
| ALT | rs10883437 | T | A | 0.64 | 0.60 |
| ALT | rs738409 | G | C | 0.23 | 0.22 |
| ALP | rs10819937 | C | G | 0.17 | 0.19 |
| ALP | rs6984305 | A | T | 0.11 | 0.12 |
| ALP | rs7186908 | C | G | 0.24 | 0.20 |
| GGT | rs2073398 | G | C | 0.34 | 0.32 |
| GGT | rs754466 | T | A | 0.24 | 0.25 |
| GGT | rs9913711 | C | G | 0.65 | 0.67 |

EAF: Effect allele frequency; ALT: alanine aminotransferase; ALP: alkaline phosphatase; GGT: gamma glutamyltransferase
