# Supplementary material for: The effect of liver enzymes on body composition: A Mendelian randomization study
Source: PLoS One. 2020 Feb 11;15(2):e0228737. doi: 10.1371/journal.pone.0228737 (PMC7012438; doi:10.1371/journal.pone.0228737)
Supplement: S4 Table — (DOCX) [file pone.0228737.s004.docx]

S4 Table. Estimates of the effect of genetically instrumented BMI^a^ on ALT, ALP, and GGT using Mendelian randomization with different methodological approaches

| Outcomes^b^ | IVW | | | WM | | MR-Egger | | | MR-PRESSO | |
| --- | --- | --- | --- | --- | --- | --- | --- | --- | --- | --- |
|  | Beta | 95% CI | I2 | Beta | 95% CI | Beta | 95% CI | Intercept p-value | Beta | 95% CI |
| ALT (U/L) | 1.39 | 0.70 to 2.08 | 77.4% | 1.28 | 0.72 to 1.84 | -0.99 | -3.59 to 1.62 | 0.06 | 1.29 | 0.80 to 1.77 |
| ALP (U/L) | 2.35 | 1.27 to 3.43 | 66.0% | 2.12 | 1.07 to 3.17 | 0.07 | -4.05 to 4.19 | 0.26 | 2.53 | 1.67 to 3.39 |
| GGT (U/L) | 0.94 | -0.74 to 2.61 | 63.6% | 1.43 | -0.23 to 3.10 | -5.89 | -12.16 to 0.38 | 0.03 | 1.29 | -0.04 to 2.63 |

SNP: single nucleotide polymorphisms; ALT: alanine aminotransferase; ALP: alkaline phosphatase; GGT: gamma glutamyltransferase

a: 96 genetically instrumented uncorrelated (R^2^<0.01) SNPs predicting BMI were obtained from the GIANT consortium (the GWAS Anthropometric 2015 BMI Summary Statistics) explaining 1.88% variance with an F statistic as 68.[1]

b: Genetic associations with ALT, ALP, and GGT were obtained from UK Biobank (~331,000 people of genetically verified white British ancestry) where the associations were obtained from multivariable linear regression adjusted for the first 20 principal components, sex, age, age-squared, the sex and age interaction and the sex and age-squared interaction.[15]
