# Supplementary material for: The effect of liver enzymes on body composition: A Mendelian randomization study
Source: PLoS One. 2020 Feb 11;15(2):e0228737. doi: 10.1371/journal.pone.0228737 (PMC7012438; doi:10.1371/journal.pone.0228737)
Supplement: Attachment — Submitted filename: Reponse to reviewers_Final.docx [file pone.0228737.s005.docx]

**Manuscript PLOS ONE**

**PONE-D-19-28757**

**Title: The effect of liver enzymes on body composition: a Mendelian randomization study**

**Responses to Editors’ and Reviewers’ comments**

# **Reviewer 1 #**

The authors examine the associations of liver enzymes with muscle and fat mass in an observational study and using Mendelian randomization (MR) approach, to test the hypothesis that liver function affects muscle mass. I have several concerns regarding the analysis.

## **C1.1**

In the Abstract (lines 15-17) the authors state that ALT “is positively associated with diabetes but inversely associated with body mass index (BMI) in Mendelian randomization (MR) studies…”  This statement seems to be based on the authors own unpublished work (ref 5, 6), as discussed in the Introduction.  I am not aware of any other (published) studies that have shown a similar relationship, thus I do not believe the relationship can be taken as a given.

*Thank you so much for this comment. Please accept our apologies for being unclear.*

*The reference 5 is our work which has been published recently indicating a negative association of ALT with BMI.[1] Instead, reference 6 is an unpublished bidirectional MR conducted by Liu ZP et al, which found an inverse association of non-alcoholic fatty liver disease (NAFLD), indicating higher level of ALT, with BMI.[2] In their case, only 2 SNPs were used to predict NAFLD. One of the SNPs used was rs738409 (PNPLA3), which strongly predicts ALT levels as well. In our published MR study,[1] we have taking the potential pleiotropy caused by NAFLD into consideration by excluding rs738409 (PNPLA3). The negative association of ALT with BMI remained.[1]* *Unfortunately, regarding the association of ALT with BMI, to our knowledge, there is no other similar MR study. However, being replicated by our two unrelated groups applying to different data sources, higher ALT is likely associated with lower BMI.*

*We have updated as below:*

***(Introduction-1^st^ paragraph)***

**From: Recently, unpublished MR studies found ALT inversely associated with body mass index (BMI), indicating higher ALT might reduce BMI.[2, 3]**

**To: Recently, ~~unpublished~~ MR studies using different data sources have found ALT inversely associated with body mass index (BMI), indicating higher ALT might reduce BMI.[1, 2]**

***(Discussion-4^th^ paragraph)***

**From: Reasons for an inverse association of ALT with fat mass are unclear but are consistent with a previous MR study~~[3]~~ showing ALT negatively associated with BMI using the same genetic variants predicting ALT applied to the 2018 GIANT and UK Biobank meta-analysis.**

**To: Reasons for an inverse association of ALT with fat mass are unclear but are consistent with a previous MR study[1] showing ALT negatively associated with BMI using the same genetic variants predicting ALT applied to the 2018 GIANT and UK Biobank meta-analysis.**

## **C1.2**

Furthermore, I find it hard to think of a plausible biological mechanism whereby liver function/liver enzymes would have a causal effect on muscle or fat-free mass.  It seems to me that an alternative explanation, that is, that decreased fat-free mass (and increased fat mass) causes NAFLD which in turn leads to elevated ALT levels, is much more plausible.  The authors should address the possibility of reverse causation (for example by performing an MR analysis to test whether genetically determined fat-free mass is associated with ALT) and should provide further support for their hypothesis (or at least provide a plausible biological explanation for the relationship).

*Thank you very much indeed for this comment.*

*With the use of MR design, it is less likely to be subjected to reverse causality, a form of confounding possibility caused by health status, given the genetic variants are randomly allocated.[4, 5] However, our study could not rule out a possibility of the causal effect of fat-free mass/fat mass on ALT via other etiological paths. As such, a bidirectional MR of liver enzymes with body composition would be more convincing. However, there was no publicly available GWAS data of liver enzymes when we conducted this study. Nevertheless, the London Life Sciences Prospective Population Study (LOLIPOPS),[6] a publicly available GWAS of liver enzymes, has been released recently. Sex-specific GWAS of liver enzymes is now available from the UK Biobank. For further interpretation, we have added MR estimates of BMI with liver enzymes (ALT, ALP, and GGT) as a supplementary file (S4 Table). We found positive associations of BMI with ALT and ALP but not GGT, which, to some extent, explains the observed positive associations of BMI with liver enzymes previously.[7-9]*

*Etiologically, an effect of ALT on fat-free mass is possibly explained by growth hormone[10-16] and sex hormones[13, 17-19], which are mainly metabolized in the liver and are associated with fat-free mass. In order to support this hypothesis, further studies should be conducted assessing the roles of liver enzymes in sex and growth hormones.*

*We have added as below:*

***(Discussion-1^st^ paragraph)***

**From: The discrepancy between the observational and MR estimates might be due to reverse causality in the cross-sectional setting and other limitations of observational studies.**

**To: The discrepancy between the observational and MR estimates might be due to ~~reverse causality in the cross-sectional setting and other limitations of observational studies.~~ the difficulty of distinguishing between cause and effect observationally. We additionally obtained an MR estimate of BMI with liver enzymes (ALT, ALP, and GGT), which gave a positive associations of BMI with ALT and ALP but not with GGT, which is consistent with the previously observed positive associations of BMI with liver enzymes.[7-9] (S4 Table).**

S4 Table Estimates of the effect of genetically instrumented BMI^a^ on ALT, ALP, and GGT using Mendelian randomization with different methodological approaches

| Outcomes^b^ | IVW | | | WM | | MR-Egger | | | MR-PRESSO | |
| --- | --- | --- | --- | --- | --- | --- | --- | --- | --- | --- |
|  | Beta | 95% CI | I2 | Beta | 95% CI | Beta | 95% CI | Intercept p-value | Beta | 95% CI |
| ALT (U/L) | 1.39 | 0.70 to 2.08 | 77.4% | 1.28 | 0.72 to 1.84 | -0.99 | -3.59 to 1.62 | 0.06 | 1.29 | 0.80 to 1.77 |
| ALP (U/L) | 2.35 | 1.27 to 3.43 | 66.0% | 2.12 | 1.07 to 3.17 | 0.07 | -4.05 to 4.19 | 0.26 | 2.53 | 1.67 to 3.39 |
| GGT (U/L) | 0.94 | -0.74 to 2.61 | 63.6% | 1.43 | -0.23 to 3.10 | -5.89 | -12.16 to 0.38 | 0.03 | 1.29 | -0.04 to 2.63 |

SNP: single nucleotide polymorphisms; ALT: alanine aminotransferase; ALP: alkaline phosphatase; GGT: gamma glutamyltransferase

a: 96 genetically instrumented uncorrelated (R^2^<0.01) SNPs predicting BMI were obtained from the GIANT consortium (the GWAS Anthropometric 2015 BMI Summary Statistics) explaining 1.88% variance with an F statistic as 68.[20]

b: Genetic associations with ALT, ALP, and GGT were obtained from UK Biobank (~331,000 people of genetically verified white British ancestry) where the associations were obtained from multivariable linear regression adjusted for the first 20 principal components, sex, age, age-squared, the sex and age interaction and the sex and age-squared interaction.[21]

***(Discussion-4^th^ paragraph)***

**From: The association of higher ALT, a measure of hepatocyte integrity, with lower fat-free mass, possibly differing by sex, may be due to growth hormone (GH) / insulin-like growth factor 1 (IGF-1) or sex hormones which are associated with chronic liver diseases and muscle mass.[10-13]**

**To: Compared with a purely observational design, MR studies are more suitable for assessing the direction of causality, given genetic variants are randomly allocated at conception.[4, 5] Etiologically, t~~T~~he association of higher ALT, a measure of hepatocyte integrity, with lower fat-free mass, possibly differing by sex, may be due to growth hormone (GH) / insulin-like growth factor 1 (IGF-1) or sex hormones which are associated with chronic liver diseases and muscle mass.[10-13]**

## **C1.3**

I am not completely clear about the hypothesis of the paper.  Do the authors suggest that liver enzymes themselves affect fat-free mass and this causes diabetes, or is it that NAFLD drives diabetes (with elevated ALT/AST levels being a proxy for NAFLD/NASH)?   If the former, then I am not convinced that all genetic variants used here are valid instruments for MR studies.  For example, the main variant used as an instrument for genetically determined ALT (PNPLA3 rs738409) is strongly associated with NAFLD, and elevated ALT is likely a consequence of hepatic steatosis.  Thus, PNPLA3 is not a valid instrument for ALT (unless the hypothesis is that NAFLD causes decreased fat-free mass, which seems to be contrary to all available evidence).  To test the hypothesis that ALT per se affects fat free mass, the authors should choose genetic instruments that are specifically associated with ALT, and not associated with NAFLD, such as variants in GPT gene (which encodes ALT).  In addition, the authors should perform a sensitivity analysis excluding PNPLA3 variant from analysis.

*Thank you very much indeed for this comment.*

*Some of the SNPs are located near the genes associated with fatty liver disease, as shown in the S2 Table. One of the 4 SNPs predicting ALT, rs738409 (PNPLA3), is associated with fatty liver disease and/or cirrhosis. Fatty liver disease is likely to be a cause of higher ALT rather than a consequence. SNPs predicting both fatty liver disease and ALT are invalid if there are other pathways by which fatty liver disease affects the outcome of interest other than via ALT. Nevertheless, the intercept terms of the MR-Egger estimates did not indicate an evidence of pleiotropy, although we cannot rule out the violation of the instrument strength independent of direct effect (InSIDE) assumption when horizontal pleiotropy of fatty liver disease causally associated with ALT.*

*We additionally assessed the association of ALT with fat-free mass, fat mass, and grip strength excluding both rs2954021 (TRIB1) (a potentially pleiotropic associated with ALP) and rs738409 (PNPLA3). The negative association of ALT with fat-free mass remained, though the confidence interval includes the null value.*

*We have added these points as below:*

***(Results-Mendelian randomization-Genetic instruments for liver enzymes-3^rd^ paragraph)***

**From: Among the 4 SNPs predicting ALT, rs2954021 (*TRIB1*) predicts both ALT and ALP.**

**To: Among the 4 SNPs predicting ALT, rs2954021 (*TRIB1*) predicts both ALT and ALP, and rs738409 (PNPLA3) is highly associated with non-alcoholic fatty liver disease**.

**(*Results Table 3 is updated as below*)**

**Table 3. Estimates of the effect of genetically instrumented ALT (per 100% change in concentration) on fat-free mass, fat mass, and grip strength (left and right) using Mendelian randomization with different methodological approaches with and without potentially pleiotropic SNPs and potentially confounded SNPs**

| Outcome | Sex | SNP^a^ | IVW | | | | WM | | MR-Egger | | | MR-PRESSO outlier corrected | | |
| --- | --- | --- | --- | --- | --- | --- | --- | --- | --- | --- | --- | --- | --- | --- |
|  |  |  | Beta | 95% CI | I2 (p-value) | Sex interaction p-value | Beta | 95% CI | Beta | 95% CI | Intercept p-value | Beta | 95% CI | Sex interaction p-value |
| Fat-free Mass (kg) | All | 4 | -0.80 | -2.41 to 0.81 | 91.4% (<0.001) | 0.68 | -0.45 | -0.98 to 0.08 | 0.73 | -2.61 to 4.07 | 0.31 | -0.41 | -0.64 to -0.19 | 0.46 |
|  |  | 3 | -0.42 | -0.91 to 0.08 | 0.0% (0.81) | 0.48 | -0.43 | -0.96 to 0.09 | -0.75 | -1.91 to 0.41 | 0.53 | - | - | - |
|  |  | 2 | -0.19 | -1.08 to 0.71 | - | 0.35 | - | - | - | - | - | - | - | - |
|  | Male | 4 | -1.10 | -3.20 to 1.00 | 84.1% (<0.001) | - | -0.74 | -1.69 to 0.21 | 0.30 | -4.61 to 5.20 | 0.53 | -0.62 | -1.49 to 0.24 | - |
|  |  | 3 | -0.62 | -1.49 to 0.25 | 0.0% (0.37) | - | -0.66 | -1.59 to 0.28 | -1.86 | -3.92 to 0.19 | 0.19 | - | - | - |
|  |  | 2 | 0.30 | -1.29 to 1.88 | - | - | - | - | - | - | - | - | - | - |
|  | Female | 4 | -0.55 | -1.87 to 0.76 | 85.3% (<0.001) | - | -0.13 | -0.70 to 0.45 | 1.08 | -1.10 to 3.25 | 0.10 | -0.25 | -0.70 to 0.20 | - |
|  |  | 3 | -0.25 | -0.77 to 0.27 | 0.0% (0.48) | - | -0.12 | -0.69 to 0.45 | 0.16 | -1.07 to 1.40 | 0.47 | - | - | - |
|  |  | 2 | -0.59 | -1.55 to 0.37 | - | - | - | - | - | - | - | - | - | - |
| Fat Mass (kg) | All | 4 | -1.22 | -3.89 to 1.46 | 93.2% (<0.001) | 0.90 | -0.59 | -1.38 to 0.19 | 1.41 | -4.02 to 6.84 | 0.28 | -0.58 | -0.85 to -0.30 | 0.57 |
|  |  | 3 | -0.58 | -1.30 to 0.15 | 0.0% (0.87) | 0.60 | -0.56 | -1.33 to 0.22 | -1.00 | -2.71 to 0.71 | 0.60 | - | - | - |
|  |  | 2 | -0.28 | -1.60 to 1.04 | - | 0.08 | - | - | - | - | - | - | - | -- |
|  | Male | 4 | -1.06 | -4.14 to 2.01 | 91.1% (<0.001) | - | -0.49 | -1.52 to 0.53 | 0.95 | -6.24 to 8.13 | 0.53 | -0.36 | -1.58 to 0.85 | - |
|  |  | 3 | -0.36 | -1.31 to 0.59 | 38.8% (0.20) | - | -0.35 | -1.38 to 0.67 | -2.24 | -4.49 to 0.01 | 0.07 | - | - | - |
|  |  | 2 | 0.95 | -0.78 to 2.68 | - | - | - | - | - | - | - | - | - | - |
|  | Female | 4 | -1.34 | -3.82 to 1.14 | 82.7% (<0.001) | - | -0.79 | -1.94 to 0.37 | 1.82 | -2.08 to 5.72 | 0.07 | -0.76 | -1.32 to -0.20 | - |
|  |  | 3 | -0.76 | -1.83 to 0.31 | 0.0% (0.76) | - | -0.73 | -1.88 to 0.42 | 0.09 | -2.43 to 2.62 | 0.47 | - | - | - |
|  |  | 2 | -1.35 | -3.30 to 0.60 | - | - | - | - | - | - | - | - | - | - |
| Left Hand Grip Strength (kg) | All | 4 | 0.00 | -0.57 to 0.57 | 0.0% (0.42) | 0.30 | -0.09 | -0.74 to 0.56 | 0.09 | -1.31 to 1.49 | 0.89 | 0.00^b^ | -0.55 to 0.55 | 0.21 |
|  |  | 3 | 0.08 | -0.52 to 0.67 | 0.0% (0.39) | 0.21 | -0.06 | -0.71 to 0.58 | -0.34 | -2.03 to 1.36 | 0.60 | - | - | - |
|  |  | 2 | 0.43 | -0.64 to 1.51 | - | 0.38 | - | - | - | - | - | - | - | - |
|  | Male | 4 | 0.33 | -0.65 to 1.32 | 0.0% (0.47) | - | 0.27 | -0.84 to 1.38 | 0.71 | -1.51 to 2.94 | 0.70 | 0.33^b^ | -0.57 to 1.23 | - |
|  |  | 3 | 0.49 | -0.53 to 1.51 | 0.0% (0.58) | - | 0.30 | -0.80 to 1.40 | -0.09 | -2.50 to 2.33 | 0.60 | - | - | - |
|  |  | 2 | 0.99 | -0.87 to 2.85 | - | - | - | - | - | - | - | - | - | - |
|  | Female | 4 | -0.30 | -0.93 to 0.34 | 0.0% (0.79) | - | -0.41 | -1.12 to 0.31 | -0.47 | -1.79 to 0.86 | 0.78 | -0.30^b^ | -0.67 to 0.08 | - |
|  |  | 3 | -0.29 | -0.95 to 0.36 | 0.0% (0.59) | - | -0.40 | -1.11 to 0.31 | -0.60 | -2.15 to 0.95 | 0.67 | - | - | - |
|  |  | 2 | -0.02 | -1.22 to 1.18 | - | - | - | - | - | - | - | - | - | - |
| Right Hand Grip Strength (kg) | All | 4 | -0.03 | -0.60 to 0.54 | 0.0% (0.64) | 0.35 | 0.06 | -0.57 to 0.70 | 0.48 | -0.71 to 1.66 | 0.34 | -0.03^b^ | -0.46 to 0.40 | 0.25 |
|  |  | 3 | 0.02 | -0.57 to 0.61 | 0.0% (0.53) | 0.20 | 0.14 | -0.51 to 0.78 | 0.47 | -0.93 to 1.86 | 0.49 | - | - | - |
|  |  | 2 | -0.25 | -1.32 to 0.83 | - | 0.54 | - | - | - | - | - | - | - | - |
|  | Male | 4 | 0.26 | -0.72 to 1.24 | 0.0% (0.48) | - | 0.49 | -0.61 to 1.58 | 1.39 | -0.66 to 3.44 | 0.22 | 0.26^b^ | -0.63 to 1.15 | - |
|  |  | 3 | 0.44 | -0.58 to 1.46 | 0.0% (0.71) | - | 0.57 | -0.53 to1.67 | 0.96 | -1.45 to 3.36 | 0.64 | - | - | - |
|  |  | 2 | 0.14 | -1.71 to 1.99 | - | - | - | - | - | - | - | - | - | - |
|  | Female | 4 | -0.31 | -0.95 to 0.33 | 0.0% (0.79) | - | -0.26 | -0.97 to 0.45 | -0.33 | -1.67 to 1.00 | 0.96 | -0.31^b^ | -0.69 to 0.07 | - |
|  |  | 3 | -0.36 | -1.02 to 0.30 | 0.0% (0.72) | - | -0.27 | -0.98 to 0.45 | 0.00 | -1.56 to 1.56 | 0.62 | - | - | - |
|  |  | 2 | -0.58 | -1.78 to 0.63 | - | - | - | - | - | - | - | - | - | - |

Potentially pleiotropic and confounded SNP: rs2954021 (*TRIB1*) **and rs738409 (*PNPLA3*)**

a SNP= 4: all SNPs; SNP= 3, excluding rs2954021**; SNP=2, excluding rs738409 additionally**

b No outlier is found, presenting the raw estimate instead

ALT: alanine aminotransferase; IVW: inverse variance weighting; WM: weighted median; MR-PRESSO: Mendelian Randomization Pleiotropy RESidual Sum and Outlier

***(Discussion-2^nd^ paragraph)***

**From: We conducted several sensitivity analyses to assess potential pleiotropy statistically, such as MR-Egger and MR-PRESSO but found no evidence of directional pleiotropy.**

**To: Pleiotropic effects are possible, but estimates remained similar after excluding potentially pleiotropic SNPs such as rs738409 (*PNPLA3*) predicting ALT. Additionally, ~~W~~we conducted several sensitivity analyses to assess potential pleiotropy statistically, such as MR-Egger and MR-PRESSO but found no evidence of directional pleiotropy.**

## **C1.4**

To investigate the relationship between liver enzymes and body composition, the authors first perform a cross-sectional analysis in a young homogeneous population “with little socioeconomic patterning of obesity…”  While this analysis may reduce the influence of confounders, it does not address the issue of reverse causality.  I.e., is it that liver enzymes affect body composition or vice versa (as described above)?

*Thank you very much indeed for this comment.*

*Although there is little evidence of socioeconomic patterning of obesity in the “Children of 1997” birth cohort, we cannot completely rule out the possibility of reverse causality in our cross-sectional design. As such, to validate the impact of liver enzymes on body composition, we conducted the MR study which is less likely to be subjected to reverse causality.[4, 5]*

*The response to this comment can also be referred to the response of C1.2*

## **C1.5**

The authors use p<0.05 as a criterion for statistical significance in their primary analysis (Results and Table 2).  However, since multiple exposures and multiple outcomes are tested, p<0.05 is not appropriate.  A more stringent significance threshold should be used to account for multiple testing and to control false positive rate.

*Thank you very much indeed for this comment. It would be informative to add the corrected p-value as a reference in interpreting the results. The positive associations of ALT with muscle mass and fat percentage, and the negative associations of ALP with muscle mass and fat percentage were robust after accounting for Boniferroni corrected p-value (<0.003, 2 exposures, 3 outcomes, and sex-specific estimates (n=3)).*

*We have added as below:*

***(Materials and methods- Statistical analyses- Observational analyses)***

**From: We assessed the associations of liver enzymes with body composition indices using multivariable linear regression, adjusted for household income, highest parental education, type of housing, highest parental occupation, second-hand and maternal smoking, height and sex.**

**To: We assessed the associations of liver enzymes with body composition indices using multivariable linear regression, adjusted for household income, highest parental education, type of housing, highest parental occupation, second-hand and maternal smoking, height and sex. Bonferroni corrected p-value (<0.003, 2 exposures, 3 outcomes, and sex-specific estimates (n=3)) was additionally used to account for multiple testing.**

***(Results- Table 2 has been updated as below)***

**Table 2. Adjusted associations of liver enzymes (ALT and ALP) with muscle mass, grip strength, and fat percentage at ~17.5 years in the Hong Kong’s “Children of 1997” birth cohort, Hong Kong, China**

| Exposure | Outcome (unit) (N) | Sex-adjusted as confounder | | P-value of interaction with sex | Boys | | Girls | |
| --- | --- | --- | --- | --- | --- | --- | --- | --- |
|  |  | Beta | 95% CI |  | Beta | 95% CI | Beta | 95% CI |
| ALT (IU/L) | Muscle mass (kg)  (2520) | 0.11^a^ | 0.10 to 0.12 | <0.001 | 0.13^a^ | 0.11 to 0.14 | 0.06^a^ | 0.04 to 0.07 |
|  | Grip strength (kg)  (2524) | 0.002 | -0.01 to 0.02 | 0.73 | 0.002 | -0.02 to 0.03 | 0.01 | -0.02 to 0.04 |
|  | Fat percentage  (2528) | 0.15^a^ | 0.13 to 0.17 | 0.19 | 0.16^a^ | 0.14 to 0.18 | 0.13^a^ | 0.09 to 0.16 |
| ALP (IU/L) | Muscle mass (kg)  (2569) | -0.03^a^ | -0.04 to -0.02 | <0.001 | -0.04^a^ | -0.05 to -0.03 | -0.005 | -0.015 to 0.005 |
|  | Grip strength (kg)  (2573) | -0.01 | -0.021 to -0.002 | 0.003 | -0.02 | -0.03 to -0.01 | 0.02 | 0.001 to 0.033 |
|  | Fat percentage  (2577) | -0.02^a^ | -0.03 to -0.01 | <0.001 | -0.03^a^ | -0.04 to -0.02 | 0.03 | 0.004 to 0.048 |

Adjustment: adjusted for household income, highest parental education, type of housing, highest parental occupation, second-hand and maternal smoking, height and sex.

ALT: alanine aminotransferase; ALP: alkaline phosphatase

**a Statistically significant in Bonferroni corrected p-value (<0.003) accounting for multiple testing**

***(Results-Children of 1997- 2^nd^ paragraph) (updates also refer to C3.7)***

**Has been updated as**

**Overall, ALT was positively associated with muscle mass (0.11, 95% CI 0.10 to 0.12) and fat percentage (0.15, 95% CI 0.13 to 0.17), whereas, ALP was negatively associated with muscle mass (-0.03, 95% CI- 0.04 to -0.02) and fat percentage (-0.02, 95% CI -0.03 to -0.01). These associations were robust to using a Bonferroni correction (p-value of 0.003). The associations of liver enzymes with muscle mass and fat percentage differed by sex (Table 2). ALT was more strongly positively associated with muscle mass (0.13, 95% CI 0.11 to 0.14) and fat percentage (0.16, 95% CI 0.14 to 0.18) in boys. ALT was not clearly associated with grip strength. ALP was inversely associated with muscle mass (-0.04, 95% CI -0.05 to -0.03), fat percentage (-0.03, 95% CI -0.04 to -0.02), and grip strength (-0.02, 95% CI -0.03 to -0.01) in boys, whereas, ALP was unclearly associated with muscle mass but positively associated with fat percentage (0.030, 95% CI 0.004 to 0.048) and grip strength (0.020, 95% CI 0.001 to 0.033) in girls.**

## **C1.6**

Table 1: I do not understand what the p-values are indicating – are these for comparison of quantitative characteristics (e.g., muscle mass) among the listed categories (e.g., male vs female)?  And how were these p-values calculated?  The statistical tests used to analyse the data should be clearly explained in the methods and/or table footnotes.  In addition, I find the table difficult to parse, since a different sample (N) is analysed for each outcome.  For clarity of presentation, and to aid the reader, the authors should use the same dataset, including complete observations (no missing outcomes or covariates) and present that in their main analysis.

*Thank you so much for this comment. Please accept our apologies for being unclear.*

*The p-values are indicating the associations of body composition (i.e., muscle mass, fat percentage, and grip strength) with potential confounders, which were assessed using independent t-tests (i.e., sex) or analysis of variance (i.e., smoking, education, occupation, income, and housing). We have added a footnote for clarification in Table 1.*

*In the main observational analyses, we only applied to the ‘complete data’ which has at least one measure of body composition. We have added the sample sizes of each specific analysis in Table 2. Additionally, given the sample size would be varied by each specific analysis, we presented the descriptive data and tested the associations of body composition indices with potential confounders in the dataset with at least one measure of body composition.*

## **C1.7**

Table 3: As discussed in comment 5 above, when testing multiple outcomes (4) using multiple methods (4), 0.05 is no longer an appropriate significance criterion.  A more stringent threshold should be used to account for multiple testing.

*Thank you very much indeed for this comment. We agree that multiple testing is a potential issue. However, we do not think that using multiple methods as sensitivity analyses constitutes multiple testing because we would not interpret a sensitivity analysis independent of the main results.*

**Minor comments:**

## **C1.8**

Line 138-139: “using inverse variance weighting (IVW) with multiplicative random effects for 4+ SNP…”  Please clarify what ‘multiplicative random effects’ means.

*Thank you so much for this comment. We are sorry for being unclear.*

*We have clarified as below:*

***(Material and methods- Statistical analyses-Mendelian randomization analyses-2^nd^ paragraph)***

**From:** **We obtained the effects of liver enzymes on body composition indices based on meta-analysis of SNP-specific Wald estimates (SNP-outcome association divided by SNP-exposure association) using inverse variance weighting (IVW) with multiplicative random effects for 4+ SNPs, which assumes balanced pleiotropy and zero average pleiotropic effect of variants, and with fixed effects for 3 SNPs or fewer.**

**To: We obtained the effects of liver enzymes on body composition indices based on meta-analysis of SNP-specific Wald estimates (SNP-outcome association divided by SNP-exposure association) using inverse variance weighting (IVW) with multiplicative random effects for 4+ SNPs, where the variance of each SNP-specific Wald estimate is multiplied by Cochran’s Q/(number of instruments minus 1) when larger than 1 to allow for heterogeneity ~~which~~ assum~~es~~ing balanced pleiotropy and zero average pleiotropic effect of variants. ~~and with~~ ~~f~~Fixed effects estimates were used for 3 SNPs or fewer. As such, both the fixed-effects and the multiplicative random-effects models give the same point estimate,[22] but usually different confidence intervals.**

## **C1.9**

Line 143: “the r^2 for genetic variant on exposure…”. Do you mean ‘the r^2 from regression of exposure on genetic variants’?

*Thank you so much for this comment. We are sorry for being unclear.*

*We have clarified as below:*

***(Material and methods- Statistical analyses-Mendelian randomization analyses-2^nd^ paragraph)***

**From:** **Power calculations were performed using the approximation that the sample size for Mendelian randomization equates to that of the same regression analysis with the sample size divided by the r^2^ for genetic variant on exposure.[23]**

**To: Power calculations were performed using the approximation that the sample size for Mendelian randomization equates to that of the same regression analysis with the sample size divided by the r^2^ ~~for~~ from regression of genetic variant on exposure.[23]**

## **C1.10**

Line 34: “to obtain providing un-confounded estimates…”  Do you mean ‘to obtain un-confounded estimates’?

*Thank you so much for this comment. We are sorry for being unclear.*

*We have revised as below:*

***(Introduction-1^st^ paragraph)***

**From:** **Mendelian randomization (MR) studies, taking advantage of the random allocation of genetic endowment at conception to obtain providing un-confounded estimates,[4] have clarified the role of liver function in T2DM.**

**To:** **Mendelian randomization (MR) studies, taking advantage of the random allocation of genetic endowment at conception to obtain ~~providing~~ un-confounded estimates,[4] have clarified the role of liver function in T2DM.**

# **Reviewer 2 #**

The authors studied the association between liver enzymes and body composition in an observational setting and Mendelian Randomization analysis. The authors provided evidence that specifically ALT was associated with a lower fat mass and fat free mass. This is an interesting, well-written and well-conducted study.
I have a few comments that need to be addressed to further improve the paper:

## **C2.1**

In both the observational and Mendelian Randomization analyses, the authors assume a linear association between the liver enzymes and the measures of body composition. However, there is increasing evidence this is not the case. For example, meta-analyses of liver enzymes and diabetes mellitus show a clear non-linear association between determinant and outcome. Is there any evidence in the current (specifically observational analyses) that the association was linear or non-linear?

*Thank you so much indeed for this helpful comment.*

*The non-linearity could not be ruled out in the case of liver enzymes related research. However, linear model of MR has been suggested valuable even if the underlying exposure-outcome association is non-linear.[24] Additionally, in our case, a two-sample MR setting, being lacking of individual data, it is less likely to account for the non-linear associations statistically,[25] unless different sets of GWAS estimates are obtained according to liver enzymes levels. In the observational design, given the participants were at their 17.5yrs, the majority were at a clinically normal range of liver enzymes levels in right-skewed distribution. As such, the statistical power of the groups located in the two tails could be a concern.*

*We have added the points as below:*

***(Discussion-1^st^ paragraph)***

**From: These two study designs have contrasting limitations.**

**To: These two study designs have contrasting limitations. In both the observational and MR designs, we assumed linear associations. Non-linearity cannot be excluded. However, observationally, most participants had clinically normal liver enzymes with a right-skewed distribution. Additionally, using a linear model in MR may be valuable even if the underlying exposure-outcome association is non-linear.[24] Individual data is needed to test for non-linear associations.**

## **C2.2**

The authors performed an observational analysis in relatively young individuals, whereas the Mendelian Randomization analysis is done in relatively older individuals from the UK Biobank population. This is difficult to compare. From literature it is known that specifically body composition at younger age has a different biological background than at older age (e.g., adult weight gain, muscle athrophy etc). The paper would benefit from a comparison of more comparable study populations (e.g., include another study of middle-aged individuals?).

*Thank you so much for this comment.*

*The observational estimates were based on the “Children of 1997” birth cohort within the participants at their 17.5yrs, which might not reflect associations in adults, given any effect determined by sex hormones might only be seen after puberty. However, almost all the participants were at least at pubertal stage 4. The variances of body compositions are likely to be different between younger age and elder age, which might be varied by physical activity, muscle gain, socioeconomic position (SEP), and ethnicity. However, more parsimoniously it is likely that the drivers of the outcomes are similar, assuming the etiological path is consistent across age and population. Nevertheless, replicating the analyses in an older cohort with little socio-economic patterning would be helpful.*

*We have addressed this point as below:*

***(Discussion- 2^nd^ paragraph)***

**From: However, we would normally expect causal factors to act consistently unless we know of reasons why the relevance of the specific operating mechanism varies by race/ethnicity.[26]**

**To: However, we would normally expect causal factors to act consistently unless we know of reasons why the relevance of the specific operating mechanism varies by race/ethnicity.[26] The age difference between the participants in the purely observational and MR designs might also contribute, as body composition may be affected by physical activity, muscle mass, and SEP. However, more parsimoniously it is likely that the drivers of the outcomes are similar, assuming the underlying etiological paths are consistent across age and population.**

## **C2.3**

The authors performed Mendelian Randomization analyses stratified by sex. However, the genetic instruments were retrieved not sex-specific. Can the authors command on this? Is there evidence that the genetic background is similar for the sexes?

*Thank you so much for this comment.*

*Given we would like to assess whether there was sex difference of the associations of liver enzymes with body composition, it would be more than informative if sex-specific estimates of the gene-exposure associations and gene-outcome associations could be utilized in the two-sample MR design. However, there was no publicly available sex-specific estimates of the liver enzymes GWAS* *when we conducted this study. As such, it might contribute to the unclearness of sex interaction in the MR design rather than in the observational design.*

*The liver is an important site for sex hormone production[27] and catabolism,[28, 29] where sex hormones are mainly synthesized and their receptors are also expressed.[30] Given the major sex hormones differ by sex, differences in liver function would be expected to have sex specific effects*

*We have added as below:*

***(Introduction- 2^nd^ paragraph)***

**From:** **To validate the impact of liver enzymes on body composition, we also used an MR design to assess the effects of genetically predicted ALT, ALP, and GGT[31] on body composition (fat-free mass, grip strength, and fat mass) from the UK Biobank.[21]**

**To: To validate the impact of liver enzymes on body composition, we also used an MR design to assess the effects of genetically predicted ALT, ALP, and GGT[31] on body composition (fat-free mass, grip strength, and fat mass) from the UK Biobank.[21]** **Differences by sex were investigated given sex disparities in liver disease[32, 33] and body composition[34] have been observed previously.**

## **C2.4**

The authors should provide more details on the used methodology to measure the liver enzymes in blood samples and how the body composition was measures. Furthermore, more details should be given on the software used for the present project.

*Thank you so much for this comment. Please accept our apologies for being unclear.*

*Observationally, we have covered the detailed measurement of the blood samples in our Biobank Clinical follow-up in the 2^nd^ paragraph of the* ***Material and methods- Observational study- the “Children of 1997” birth cohort*** *section. In the MR design, there is no consistent detailed measurement could be accessed, given the GWAS estimates were drawn from different cohorts or different centers.*

*We have also added theses points as below:*

*(****Material and methods- Observational study- the “Children of 1997” birth cohort- 2^nd^ paragraph)***

**From: Body composition indices including muscle mass and fat percentage were measured using bioimpedance analysis by a Tanita segmental body composition monitor (Tanita BC-545, Tanita Co., Tokyo, Japan). Grip strength was measured by the Takei T.K.K.5401 GRIP D handgrip dynamometer (Takei Scientific Instruments Co. Ltd, Tokyo, Japan).**

**To: Body composition indices including muscle mass and fat percentage were measured using bioimpedance analysis by a Tanita segmental body composition monitor (Tanita BC-545, Tanita Co., Tokyo, Japan). Grip strength was measured by the Takei T.K.K.5401 GRIP D handgrip dynamometer (Takei Scientific Instruments Co. Ltd, Tokyo, Japan). All anthropometric measurements were made by trained research assistants following specific standard protocols.**

## **C2.5**

For comparison of the observational and MR findings, it would be good that both analyses are performed in the same unit. The GWAS (and thus the unit of the MR analysis) was log transformed.

*Thank you so much for this comment. We are so sorry that we cannot present the consistent unit in both the observational and MR estimates, though we had accounted for this important issue. Because there was not much information could be obtained from original GWAS of liver enzymes, converting the 100% change unit into,. e.g., a standard unit was not available.*

*We have added as below:*

***(Material and methods-Mendelian randomization- Exposure - genetic predictors of liver enzymes)***

**From:** **Single nucleotide polymorphisms (SNPs) predicting plasma log-transformed ALT, ALP and GGT at genome-wide significance (p-value<5×10^-8^) adjusted for age and sex were obtained from the largest available genome-wide association study (GWAS) of plasma levels of liver enzymes comprising 61,089 adults (~86% European, mean age 52.8 years, 50.6% women).[31]**

**To:** **Single nucleotide polymorphisms (SNPs) predicting plasma log-transformed ALT, ALP and GGT at genome-wide significance (p-value<5×10^-8^) adjusted for age and sex were obtained from the largest available genome-wide association study (GWAS) of plasma levels of liver enzymes comprising 61,089 adults (~86% European, mean age 52.8 years, 50.6% women). The estimate for each SNP obtained from the GWAS represents the % change in concentration of liver enzyme (effect size) in plasma per copy of the effect allele.[31]**

## **C2.6**

Line 129: "small proportion". Can the authors provide a percentage?

*Thank you so much for this comment. Please accept our apologies for being unclear.*

*We have added as below:*

*(****Material and methods- Statistical analyses- Observational analyses)***

**From: For a small proportion of the observations, ALT was lower than 10 IU/L (n=254), without specific value, were fixed at 5 IU/L.**

**To: For a small proportion of the observations, ALT was lower than 10 IU/L (n=254, 7.3%), without specific value, were fixed at 5 IU/L.**

## **C2.7**

The authors might was to think of a different way to present the data. Given the very small effect sizes, results are hard to interpret.

*Thank you very much for this comment.*

*Clinically, abnormal liver function is usually indicated by 2-3 times or more elevated liver enzymes,[35] which is equal to 200% - 300% elevated of the base levels, although, in some case, abnormal results may not be seen until the development of a significant liver disease.[36] The unit of the MR estimates is kg change per 100% elevation of liver enzymes level, in another word, the unit we present is not that small. Additionally, regarding the normal range, from a public health perspective even relatively small differences can make a difference to the population.*

## **C2.8**

The conclusion of the manuscript is heavily weighted on the fat-free mass findings, but do not take into account the additional findings with fat mass. The original aim was that the previously observed association between ALT and BMI will be further explored using fat mass and fat-free mass. However, the authors should note that both outcomes show rather similar results.

*Thank you very much indeed for this comment.*

*This MR study provided an indication that higher ALT was associated with lower fat mass, which, to some extent, could explain the previous findings that the negative association of ALT with BMI in the MR studies.[1, 2] However, etiologically, we would like to address the potential paths of the positive association of ALT with diabetes risk,[37, 38] which has been supported by different researchers using MR design. Given fat mass and adiposity are well-established causal factor of diabetes*,*[39-41] it is less likely contributing to the positive association of ALT with diabetes in this scenario. As such less attention were addressed to this finding,*

*We have added as below:*

***(Discussion-4^th^ paragraph)***

**From: Reasons for an inverse association of ALT with fat mass are unclear but are consistent with a previous MR study[1] showing ALT negatively associated with BMI using the same genetic variants predicting ALT applied to the 2018 GIANT and UK Biobank meta-analysis.**

**To: Reasons for an inverse association of ALT with fat mass are unclear since fat mass is a well-established causal factor for diabetes which is unlikely to be contributing to the positive association of ALT with diabetes seen here,[1, 2] ~~b~~But these inverse estimates are consistent with a previous MR study[1] showing ALT negatively associated with BMI using the same genetic variants predicting ALT applied to the 2018 GIANT and UK Biobank meta-analysis.**

## **C2.9**

The authors should acknowledge the fact the ALT has a limited number of instruments for the MR analyses (specifically after excluding potential pleiotropic SNPs).

*Thank you very much for this comment.*

*We have added as below:*

***(Discussion- 2^nd^ paragraph)***

**From: We conducted several sensitivity analyses to assess potential pleiotropy statistically, such as MR-Egger and MR-PRESSO but found no evidence of directional pleiotropy.**

**To: We conducted several sensitivity analyses to assess potential pleiotropy statistically, such as MR-Egger and MR-PRESSO but found no evidence of directional pleiotropy. Given only 4 SNPs predicted ALT, excluding potentially pleiotropic and confounded SNPs reduce statistical power.**

# **Reviewer 3 #**

Liu XJ et al. assessed the associations of liver enzymes with muscle and fat mass using a two-sample MR. Authors find that higher ALT could reduce fat-free mass and fat mass, while higher GGT, as a might increase fat-free mass and fat mass. Also, authors report sex differences in the association of markers of liver functions and body composition.

## **C3.1**

Do authors have information on cardiovascular risk factors such as blood pressure, glucose and blood lipids? If yes, then authors should consider adjusting for these factors.

*Thank you very much for this comment.*

*Observationally, information on cardiovascular risk factors are available in the “Children of 1997” birth cohort. However, these covariates are less likely to be considered as potential confounders since there is no strong evidence indicating the cardiovascular risk factors cause fat-free mass and fat mass. Being analogous to the MR design, cardiovascular risk factors were not considered as potential confounders as well when checking and excluding potential confounded SNPs. As such, we did not take these cardiovascular risk factors into account in the analyses.*

## **C3.2**

In the observational study authors should adjust for smoking and alcohol intake. A sensitivity analysis should be performed among non-smokers and non-drinkers.

*Thank you for this comment.*

*Unfortunately, we have no information of smoking and alcohol intake in the Biobank Clinical follow-up of the “Children of 1997” birth cohort. So that conducting such an adjustment is not available. However, smoking and alcohol intake are less likely to be potential confounders in the “Children of 1997” birth cohort. Because, smoking is rare (i.e., 1% cigarette smokers among Hong Kong teenagers at 15-19 in 2018; 2.7% current smokers among secondary students in 2014/2015) and alcohol consumption is low (the prevalence of past 30-day alcohol drinking in Hong Kong adolescents was 18.7% in 2011/2012) among Hong Kong adolescents.[42-44] Additionally, with limited number of smoker and drinkers, there might not be enough statistical power to address these concerns.*

## **C3.3**

Authors select the SNPs based on Caucasians, while the observational association is explored in Chinese population. Liver function and obesity differs by ethnicity; do authors have an explanation for their choice? Did they test the association of selected SNPs with ALT/AST/GGT in Asian population? Is there any published GWAS on markers of liver function in Asian population?

*Thank you very much for this comment.*

*The external validity and generalizability are concerns when comparing these two study designs applying to different population. Findings in a Chinese population might not be relevant to other populations. External validity, i.e., generalizing or transporting from a subgroup to the full target population or another population of interest, is always a concern in epidemiological studies.[26, 45-47] The key issue of external validity is how the exposure works, so as to be able to assess whether the mechanism is relevant in a new population.[26] We would normally expect causal factors to act consistently unless we know of reasons why the relevance of the specific operating mechanism varies by race/ethnicity.[26]*

*The MR study mainly pertained to people of European ancestry. However, again the key issue is how the exposure works and whether it would be relevant in a new population. There is no reason to expect liver function to have different effects in different populations, however, the possibility cannot be completely excluded. As such, replication studies in people of different ancestry and different age groups would be valuable. However, large open-access GWAS of Chinese people do not exist, although some GWAS of Japanese people are available. Discrepancies between the observational estimates and the MR estimates might be due to the differences in ethnicity, which could not be ruled out in the lack of available data, although there is no reason to think liver function has different effects by ethnicity.*

*We have added as below:*

***(Discussion- 2^nd^ paragraph)***

**From: Replicating the MR study in a Chinese population would be very helpful.**

**To: Replicating the MR study in a Chinese population would be very helpful. However, publicly available large GWAS of liver enzymes or body composition in Chinese does not exist.**

## **C3.4**

Was there information on medications that could alter liver functions? These should be considered in the observational analysis.

*Thank you very much for this comment.*

*We are sorry that there is no information on medication usage in the Biobank Clinical follow-up of the “Children of 1997” birth cohort. Medication usage can be regarded as a potential confounder when addressing the questions of liver enzymes related studies. However, the participants in the Biobank Clinical follow-up of the “Children of 1997” birth cohort were at their age of 17.5yrs. The majority of them were at the normal range of liver enzymes. Medication usage is rare. As such, confounding bias caused by medication usage was less likely albeit it cannot be completely ruled out.*

*We have added as below:*

***(Discussion- 2^nd^ paragraph)***

**From:** **Observational studies are open to residual confounding, possibly by diet, lifestyle, and physical activity, although smoking is rare and alcohol consumption is low in Hong Kong adolescents.[42-44]**

**To: Observational studies are open to residual confounding, possibly by diet, medication usage, lifestyle, and physical activity, although medication use is rare at 17.5 years and specifically in Hong Kong adolescents, while smoking is rare and alcohol consumption is low ~~in Hong Kong adolescents~~.[42-44]**

## **C3.5**

Authors should discuss further what could the reason of the sex-differences they find.
A previous MR study reported no causal effect of GGT on prediabetes and diabetes (Nano et al. 2017) in both men and women. In the current study, there is a suggestive causal effect of GGT on fat mass, particularly in women. What would be the implications of such findings?

*Thank you so much for this comment.*

*Considering sex hormones are mainly synthesized in liver[30] and endogenous sex hormones are associated with body composition,[17-19] we hypothesized that poorer liver function, mainly indicated by higher ALT, drives an association of lower sex hormones[13] with higher diabetes risk mediated by muscle mass. Sex hormones could be potential mediators because they are associated with liver diseases and muscle mass.[13, 17-19] As such, we assessed whether associations differed by sex from the relevant interaction terms after adjusting for potentially confounding interactions observationally.*

*In the discussion section, we did not elaborate in depth considering to the issue of over-interpretation. Instead, we proposed growth hormone (GH) / insulin-like growth factor 1 (IGF-1) or sex hormones as potentially etiological paths, which have been supported by animal[14-16] and population evidence.[12, 13]*

*Our previous MR study also suggested no causal role of GGT on diabetes but indicated a positive association of GGT with cardiovascular disease risk, although the confidence interval included the null value.[37] The positive associations of GGT with fat mass might indicate potential paths of the observed association of GGT with cardiovascular disease risks.[48-50] However, the positive estimates were not robust enough to draw too much attention in this manuscript only.*

*We have added as below:*

***(Discussion- 4^th^ paragraph)***

**From: But the inverse estimates is consistent with a previous MR study[1] showing ALT negatively associated with BMI using the same genetic variants predicting ALT applied to the 2018 GIANT and UK Biobank meta-analysis.**

**To: But the inverse estimates is consistent with a previous MR study[1] showing ALT negatively associated with BMI using the same genetic variants predicting ALT applied to the 2018 GIANT and UK Biobank meta-analysis. The positive associations of GGT with body composition, in particular with fat mass, might be relevant to the observed association of GGT with cardiovascular disease risk.[48-50] However, no causal role of GGT in cardiovascular disease was found in an MR study.[37]**

## **C3.6**

In the observational study authors do not investigate the association between GGT and body composition parameters, which limits their MR findings.

*Thank you so much for this comment.*

*Unfortunately, there was no GGT measurement in the Biobank Clinical follow-up of the “Children of 1997” birth cohort. Considering a compromise between cost and comprehensiveness, liver function was only assessed from ALT and ALP.*

**Minor comments**

## **C3.7**

When describing the results, authors should provide estimates and not just reporting there was or not an association.

*Thank you very much for your comments.*

*Observationally, we have updated the* ***Results*** *section which could be referred to the Response of C1.5. The description of Results section of the MR estimates is updated as below:*

***(Results-Mendelian randomization)***

**Be updated as**

**Table 3 shows similar inverse estimates of genetically predicted ALT with fat-free mass and fat mass from all methods and by sex, however, the confidence intervals included the null value. ALT was not clearly associated with grip strength. Nevertheless, using MR-PRESSO ALT was inversely associated with fat-free mass (-0.41, 95% CI -0.64 to -0.19) and fat mass (-0.58, 95% CI -0.85 to -0.30).**

**Table 5 shows genetically predicted GGT was not clearly associated with fat-free mass, fat mass or grip strength, but after excluding potential pleiotropy the corrected MR-PRESSO estimates suggested a positive association with fat-free mass (0.30, 95% CI 0.01 to 0.60) and fat mass (0.41, 95% CI 0.10 to 0.71), particularly in women. GGT was not clearly associated with grip strength, although the WM estimate gave positive associations in women.**

## **C3.8**

The manuscripts needs English Editing. Also in the method section, authors should describe first the observational association and then the MR.

*Thank you very much for your comments. Please accept our apologise for the unclear layout.*

*In the whole manuscript including* ***the Method and material*** *section, and the* ***Statistical analyses*** *section, we list the observational part before the MR section consistently.*

*Regarding English editing, We have reviewed the English throughout.*

**Reference**:

1.	Liu J, Au Yeung SL, Kwok MK, Leung JYY, Lin SL, Hui LL, et al. The effect of liver enzymes on adiposity: a Mendelian randomization study. Sci Rep. 2019;9(1):16792-. doi: 10.1038/s41598-019-52489-8. PubMed PMID: 31727910.

2.	Liu Z, Zhang Y, Graham S, Pique-Regi R, Dong XC, Chen YE, et al. Mendelian Randomization Analysis Dissects the Relationship between NAFLD, T2D, and Obesity and Provides Implications to Precision Medicine. bioRxiv. 2019:657734. doi: 10.1101/657734.

3.	Liu JX, Au Yeung SL, Kwok MK, Yan Leung JY, Lin SL, Hui LL, et al. The effect of liver enzymes on adiposity: a Mendelian randomization study. bioRxiv. 2018:404319. doi: 10.1101/404319.

4.	Lawlor DA, Harbord RM, Sterne JA, Timpson N, Davey Smith G. Mendelian randomization: using genes as instruments for making causal inferences in epidemiology. Statistics in medicine. 2008;27(8):1133-63. doi: 10.1002/sim.3034. PubMed PMID: 17886233.

5.	Davies NM, Holmes MV, Davey Smith G. Reading Mendelian randomisation studies: a guide, glossary, and checklist for clinicians. BMJ. 2018;362:k601. doi: 10.1136/bmj.k601.

6.	The London Life Sciences Prospective Population Study. Available from: <http://www.lolipopstudy.org/>.

7.	Rinella ME. Nonalcoholic fatty liver disease: a systematic review. Jama. 2015;313(22):2263-73. Epub 2015/06/10. doi: 10.1001/jama.2015.5370. PubMed PMID: 26057287.

8.	Fan JG, Kim SU, Wong VW. New trends on obesity and NAFLD in Asia. Journal of hepatology. 2017;67(4):862-73. Epub 2017/06/24. doi: 10.1016/j.jhep.2017.06.003. PubMed PMID: 28642059.

9.	Di Domizio S, Moscatiello S, Marchesini G, Forlani G. Obesity-Associated Liver Disease. The Journal of Clinical Endocrinology & Metabolism. 2008;93(11_supplement_1):s74-s80. doi: 10.1210/jc.2008-1399.

10.	Guichelaar MM, Charlton MR. Decreased muscle mass in nonalcoholic fatty liver disease: new evidence of a link between growth hormone and fatty liver disease? Hepatology (Baltimore, Md). 2014;59(5):1668-70. Epub 2014/04/03. doi: 10.1002/hep.27058. PubMed PMID: 24691865.

11.	Cabrera D, Ruiz A, Cabello-Verrugio C, Brandan E, Estrada L, Pizarro M, et al. Diet-Induced Nonalcoholic Fatty Liver Disease Is Associated with Sarcopenia and Decreased Serum Insulin-Like Growth Factor-1. Digestive diseases and sciences. 2016;61(11):3190-8. Epub 2016/10/19. doi: 10.1007/s10620-016-4285-0. PubMed PMID: 27572941.

12.	Schooling CM, Au Yeung SL, Leung GM. Why do statins reduce cardiovascular disease more than other lipid modulating therapies? Eur J Clin Invest. 2014;44(11):1135-40. Epub 2014/09/25. doi: 10.1111/eci.12342. PubMed PMID: 25252212.

13.	Jaruvongvanich V, Sanguankeo A, Riangwiwat T, Upala S. Testosterone, Sex Hormone-Binding Globulin and Nonalcoholic Fatty Liver Disease: a Systematic Review and Meta-Analysis. Annals of hepatology. 2017;16(3):382-94. Epub 2017/04/21. doi: 10.5604/16652681.1235481. PubMed PMID: 28425408.

14.	Sjogren K, Wallenius K, Liu JL, Bohlooly YM, Pacini G, Svensson L, et al. Liver-derived IGF-I is of importance for normal carbohydrate and lipid metabolism. Diabetes. 2001;50(7):1539-45. Epub 2001/06/26. PubMed PMID: 11423474.

15.	Yakar S, Liu JL, Fernandez AM, Wu Y, Schally AV, Frystyk J, et al. Liver-specific igf-1 gene deletion leads to muscle insulin insensitivity. Diabetes. 2001;50(5):1110-8. Epub 2001/05/04. PubMed PMID: 11334415.

16.	Sandhu MS. Insulin-like growth factor-I and risk of type 2 diabetes and coronary heart disease: molecular epidemiology. Endocrine development. 2005;9:44-54. Epub 2005/05/10. doi: 10.1159/000085755. PubMed PMID: 15879687.

17.	Ng Tang Fui M, Hoermann R, Zajac JD, Grossmann M. The effects of testosterone on body composition in obese men are not sustained after cessation of testosterone treatment. Clinical endocrinology. 2017;87(4):336-43. Epub 2017/06/01. doi: 10.1111/cen.13385. PubMed PMID: 28561278.

18.	Ng Tang Fui M, Prendergast LA, Dupuis P, Raval M, Strauss BJ, Zajac JD, et al. Effects of testosterone treatment on body fat and lean mass in obese men on a hypocaloric diet: a randomised controlled trial. BMC medicine. 2016;14(1):153. Epub 2016/10/08. doi: 10.1186/s12916-016-0700-9. PubMed PMID: 27716209; PubMed Central PMCID: PMCPMC5054608.

19.	Sinclair M, Grossmann M, Hoermann R, Angus PW, Gow PJ. Testosterone therapy increases muscle mass in men with cirrhosis and low testosterone: A randomised controlled trial. Journal of hepatology. 2016;65(5):906-13. Epub 2016/10/19. doi: 10.1016/j.jhep.2016.06.007. PubMed PMID: 27312945.

20.	Locke AE, Kahali B, Berndt SI, Justice AE, Pers TH, Day FR, et al. Genetic studies of body mass index yield new insights for obesity biology. Nature. 2015;518(7538):197-206. Epub 2015/02/13. doi: 10.1038/nature14177. PubMed PMID: 25673413; PubMed Central PMCID: PMCPMC4382211.

21.	Howrigan D. DETAILS AND CONSIDERATIONS OF THE UK BIOBANK GWAS: THE NEALE LAB; September 20, 2017. Available from: <http://www.nealelab.is/blog/2017/9/11/details-and-considerations-of-the-uk-biobank-gwas>.

22.	Rees JMB, Wood AM, Burgess S. Extending the MR-Egger method for multivariable Mendelian randomization to correct for both measured and unmeasured pleiotropy. Statistics in medicine. 2017;36(29):4705-18. Epub 2017/09/30. doi: 10.1002/sim.7492. PubMed PMID: 28960498; PubMed Central PMCID: PMCPMC5725762.

23.	Freeman G, Cowling BJ, Schooling CM. Power and sample size calculations for Mendelian randomization studies using one genetic instrument. International journal of epidemiology. 2013;42(4):1157-63. Epub 2013/08/13. doi: 10.1093/ije/dyt110. PubMed PMID: 23934314.

24.	Angrist JD, Pischke J-S. The Credibility Revolution in Empirical Economics: How Better Research Design Is Taking the Con out of Econometrics. Journal of Economic Perspectives. 2010;24(2):3-30. doi: 10.1257/jep.24.2.3.

25.	Burgess S, Davies NM, Thompson SG. Instrumental variable analysis with a nonlinear exposure-outcome relationship. Epidemiology. 2014;25(6):877-85. Epub 2014/08/29. doi: 10.1097/ede.0000000000000161. PubMed PMID: 25166881; PubMed Central PMCID: PMCPMC4222800.

26.	Lopez P, Subramanian SV, Schooling CM. Effect measure modification conceptualized using selection diagrams as medication by mechanisms of varying population-level relevance. J Clin Epidemiol. 2019. Epub 2019/05/24. doi: 10.1016/j.jclinepi.2019.05.005. PubMed PMID: 31121303.

27.	Tsuchiya Y, Nakajima M, Yokoi T. Cytochrome P450-mediated metabolism of estrogens and its regulation in human. Cancer letters. 2005;227(2):115-24. Epub 2005/08/23. doi: 10.1016/j.canlet.2004.10.007. PubMed PMID: 16112414.

28.	Chouinard S, Yueh MF, Tukey RH, Giton F, Fiet J, Pelletier G, et al. Inactivation by UDP-glucuronosyltransferase enzymes: the end of androgen signaling. The Journal of steroid biochemistry and molecular biology. 2008;109(3-5):247-53. Epub 2008/05/10. doi: 10.1016/j.jsbmb.2008.03.016. PubMed PMID: 18467088.

29.	Raftogianis R, Creveling C, Weinshilboum R, Weisz J. Chapter 6: Estrogen Metabolism by Conjugation. JNCI Monographs. 2000;2000(27):113-24.

30.	Pelletier G. Localization of androgen and estrogen receptors in rat and primate tissues. Histology and histopathology. 2000;15(4):1261-70. Epub 2000/09/27. PubMed PMID: 11005250.

31.	Chambers JC, Zhang W, Sehmi J, Li X, Wass MN, Van der Harst P, et al. Genome-wide association study identifies loci influencing concentrations of liver enzymes in plasma. Nature genetics. 2011;43(11):1131-8. Epub 2011/10/18. doi: 10.1038/ng.970. PubMed PMID: 22001757; PubMed Central PMCID: PMCPMC3482372.

32.	Lonardo A, Nascimbeni F, Ballestri S, Fairweather D, Win S, Than TA, et al. Sex Differences in Nonalcoholic Fatty Liver Disease: State of the Art and Identification of Research Gaps. Hepatology (Baltimore, Md). 2019;70(4):1457-69. doi: 10.1002/hep.30626.

33.	Ferlay J, Colombet M, Soerjomataram I, Mathers C, Parkin DM, Pineros M, et al. Estimating the global cancer incidence and mortality in 2018: GLOBOCAN sources and methods. Int J Cancer. 2019;144(8):1941-53. Epub 2018/10/24. doi: 10.1002/ijc.31937. PubMed PMID: 30350310.

34.	Bredella MA. Sex Differences in Body Composition. Adv Exp Med Biol. 2017;1043:9-27. Epub 2017/12/11. doi: 10.1007/978-3-319-70178-3_2. PubMed PMID: 29224088.

35.	Newsome PN, Cramb R, Davison SM, Dillon JF, Foulerton M, Godfrey EM, et al. Guidelines on the management of abnormal liver blood tests. Gut. 2018;67(1):6. doi: 10.1136/gutjnl-2017-314924.

36.	Williams R, Aspinall R, Bellis M, Camps-Walsh G, Cramp M, Dhawan A, et al. Addressing liver disease in the UK: a blueprint for attaining excellence in health care and reducing premature mortality from lifestyle issues of excess consumption of alcohol, obesity, and viral hepatitis. Lancet (London, England). 2014;384(9958):1953-97. Epub 2014/12/01. doi: 10.1016/s0140-6736(14)61838-9. PubMed PMID: 25433429.

37.	Liu J, Au Yeung SL, Lin SL, Leung GM, Schooling CM. Liver Enzymes and Risk of Ischemic Heart Disease and Type 2 Diabetes Mellitus: A Mendelian Randomization Study. Scientific reports. 2016;6:38813. Epub 2016/12/21. doi: 10.1038/srep38813. PubMed PMID: 27996050; PubMed Central PMCID: PMCPMC5171875.

38.	De Silva NMG, Borges MC, Hingorani A, Engmann J, Shah T, Zhang X, et al. Liver Function and Risk of Type 2 Diabetes: Bidirectional Mendelian Randomization Study. Diabetes. 2019:db181048. doi: 10.2337/db18-1048.

39.	Swerdlow DI. Mendelian Randomization and Type 2 Diabetes. Cardiovascular Drugs and Therapy. 2016;30:51-7. doi: 10.1007/s10557-016-6638-5. PubMed PMID: PMC4789194.

40.	Frayling TM, Stoneman CE. Mendelian randomisation in type 2 diabetes and coronary artery disease. Current opinion in genetics & development. 2018;50:111-20. Epub 2018/06/24. doi: 10.1016/j.gde.2018.05.010. PubMed PMID: 29935421.

41.	Riaz H, Khan MS, Siddiqi TJ, Usman MS, Shah N, Goyal A, et al. Association Between Obesity and Cardiovascular Outcomes: A Systematic Review and Meta-analysis of Mendelian Randomization Studies. JAMA Network Open. 2018;1(7):e183788-e. doi: 10.1001/jamanetworkopen.2018.3788.

42.	Statistics on Youth Health-related Behaviour: Centre for Health Protection Department of Health The Government of Hong Kong Special Administrative Region; [updated 2018/11/08; cited 2019]. Available from: <https://www.chp.gov.hk/en/statistics/data/10/757/5522.html>.

43.	Au WM, Ho SY, Wang MP, Lo WS, Tin SPP, Huang R, et al. Alcohol Drinking and Pro-drinking Practices in Parents of Hong Kong Adolescents. Alcohol and Alcoholism. 2014;49(6):668-74. doi: 10.1093/alcalc/agu063.

44.	HKSAR HKCaSDo. Thematic Household Survey Report No.64. In: HKSAR HKCaSDo, editor. 2018.

45.	Cole SR, Stuart EA. Generalizing Evidence From Randomized Clinical Trials to Target Populations: The ACTG 320 Trial. American journal of epidemiology. 2010;172(1):107-15. doi: 10.1093/aje/kwq084.

46.	Lesko CR, Buchanan AL, Westreich D, Edwards JK, Hudgens MG, Cole SR. Generalizing Study Results: A Potential Outcomes Perspective. Epidemiology (Cambridge, Mass). 2017;28(4):553-61. doi: 10.1097/ede.0000000000000664. PubMed PMID: 00001648-201707000-00013.

47.	Bareinboim E, Pearl J. Causal inference and the data-fusion problem. Proc Natl Acad Sci U S A. 2016;113(27):7345-52. Epub 2016/07/07. doi: 10.1073/pnas.1510507113. PubMed PMID: 27382148; PubMed Central PMCID: PMCPMC4941504.

48.	Meisinger C, Doring A, Schneider A, Lowel H. Serum gamma-glutamyltransferase is a predictor of incident coronary events in apparently healthy men from the general population. Atherosclerosis. 2006;189(2):297-302. Epub 2006/02/18. doi: 10.1016/j.atherosclerosis.2006.01.010. PubMed PMID: 16483579.

49.	Fraser A, Harris R, Sattar N, Ebrahim S, Smith GD, Lawlor DA. Gamma-glutamyltransferase is associated with incident vascular events independently of alcohol intake: analysis of the British Women's Heart and Health Study and Meta-Analysis. Arteriosclerosis, thrombosis, and vascular biology. 2007;27(12):2729-35. Epub 2007/10/13. doi: 10.1161/ATVBAHA.107.152298. PubMed PMID: 17932318.

50.	Ghouri N, Preiss D, Sattar N. Liver enzymes, nonalcoholic fatty liver disease, and incident cardiovascular disease: a narrative review and clinical perspective of prospective data. Hepatology (Baltimore, Md). 2010;52(3):1156-61. Epub 2010/07/27. doi: 10.1002/hep.23789. PubMed PMID: 20658466.
